# Photodynamic Therapy—Current Limitations and Novel Approaches

**DOI:** 10.3389/fchem.2021.691697

**Published:** 2021-06-10

**Authors:** Gurcan Gunaydin, M. Emre Gedik, Seylan Ayan

**Affiliations:** ^1^ Department of Basic Oncology, Hacettepe University Cancer Institute, Sihhiye, Ankara, Turkey; ^2^ Department of Chemistry, Bilkent University, Ankara, Turkey

**Keywords:** photodynamic therapy, tumor, photosensitizer, current limitations, novel approaches, bioengieering, selectivity, targeting

## Abstract

Photodynamic therapy (PDT) mostly relies on the generation of singlet oxygen, via the excitation of a photosensitizer, so that target tumor cells can be destroyed. PDT can be applied in the settings of several malignant diseases. In fact, the earliest preclinical applications date back to 1900’s. Dougherty reported the treatment of skin tumors by PDT in 1978. Several further studies around 1980 demonstrated the effectiveness of PDT. Thus, the technique has attracted the attention of numerous researchers since then. Hematoporphyrin derivative received the FDA approval as a clinical application of PDT in 1995. We have indeed witnessed a considerable progress in the field over the last century. Given the fact that PDT has a favorable adverse event profile and can enhance anti-tumor immune responses as well as demonstrating minimally invasive characteristics, it is disappointing that PDT is not broadly utilized in the clinical setting for the treatment of malignant and/or non-malignant diseases. Several issues still hinder the development of PDT, such as those related with light, tissue oxygenation and inherent properties of the photosensitizers. Various photosensitizers have been designed/synthesized in order to overcome the limitations. In this Review, we provide a general overview of the mechanisms of action in terms of PDT in cancer, including the effects on immune system and vasculature as well as mechanisms related with tumor cell destruction. We will also briefly mention the application of PDT for non-malignant diseases. The current limitations of PDT utilization in cancer will be reviewed, since identifying problems associated with design/synthesis of photosensitizers as well as application of light and tissue oxygenation might pave the way for more effective PDT approaches. Furthermore, novel promising approaches to improve outcome in PDT such as selectivity, bioengineering, subcellular/organelle targeting, etc. will also be discussed in detail, since the potential of pioneering and exceptional approaches that aim to overcome the limitations and reveal the full potential of PDT in terms of clinical translation are undoubtedly exciting. A better understanding of novel concepts in the field (*e.g.* enhanced, two-stage, fractional PDT) will most likely prove to be very useful for pursuing and improving effective PDT strategies.

## Introduction

Photodynamic therapy (PDT) is a therapeutic modality for specific malignant (*e.g.* gastrointestinal, skin, head and neck, and gynecological cancers) as well as non-malignant [*e.g.* age related macular degeneration (AMD), psoriasis] and pre-malignant (*e.g.* actinic keratosis) conditions (Brown et al., 2004; Castano et al., 2006b). The mechanisms of action depend on the generation of singlet oxygen (^1^O_2_), preferentially with high yield, through the excitation of a particular photosensitizer (PS), which transfers its excited energy to the molecular oxygen in tumor tissues *via* triplet state manifold. The necrotic and/or apoptotic destruction of the tumor cells are induced by cytotoxic singlet oxygen and other secondary molecules such as reactive oxygen species (ROS). Detailed mechanisms of action of PDT will be summarized in the next section. The earliest preclinical applications of PDT were published around 1900’s. Raab showed the dependence of light and the necessity that the light be of wavelengths that were absorbed by the sensitizing “dye” (Raab, 1900). Von Tappeiner eventually coined the term “photodynamic” to refer to this photosensitization. Von Tappeiner applied eosin to basal cell carcinomas topically, followed by the application of visible light to the region. The results demonstrated that target tissue destruction was achieved by the dynamic interaction among light, oxygen and the photosensitizing agent (von Tappeiner and Jodlbauer, 1907). In 1995, Photofrin [commercial name for HpD (hematoporphyrin derivative)] received the FDA approval as a clinical application of PDT by the efforts of Dougherty (Dougherty et al., 1998). Eventually, the true potential of PDT for therapeutic applications against tumor tissues had been recognized more than 70 years after Raab’s studies. HpD had some limitations, since it had a weak absorption band in the near-infrared (NIR) region, rendering it not suitable for deep-seated tumor tissues. As a result, second generation of PSs [*e.g.* chlorins, benzoporphyrin derivatives, texaphyrins, phthalocyanines and natural products such as hypericin and protoporphyrin IX (PpIX)] were designed in order to overcome the limitations of HpD. These second generation PSs have been used in cardiovascular and ophthalmological diseases (Rockson et al., 2000; Sivaprasad and Hykin, 2006). The third generation PSs integrated strategies that utilized targeting (active and passive targeting) and delivery moieties, such as monoclonal antibodies, high-affinity ligands (*e.g.* peptides, antibodies, nucleic acids, vitamins or carbohydrates) that can be attached to nanoparticles (NPs), liposomes and ligands targeting overexpressed receptors (Chen et al., 2010). The current limitations of PDT applications will be discussed in detail in this review. Furthermore, novel promising approaches in terms of PDT will also be reviewed, since the potential of pioneering approaches in order to overcome such limitations of PDT are truly exciting.

## Mechanisms of Action

**FIGURE 1:**
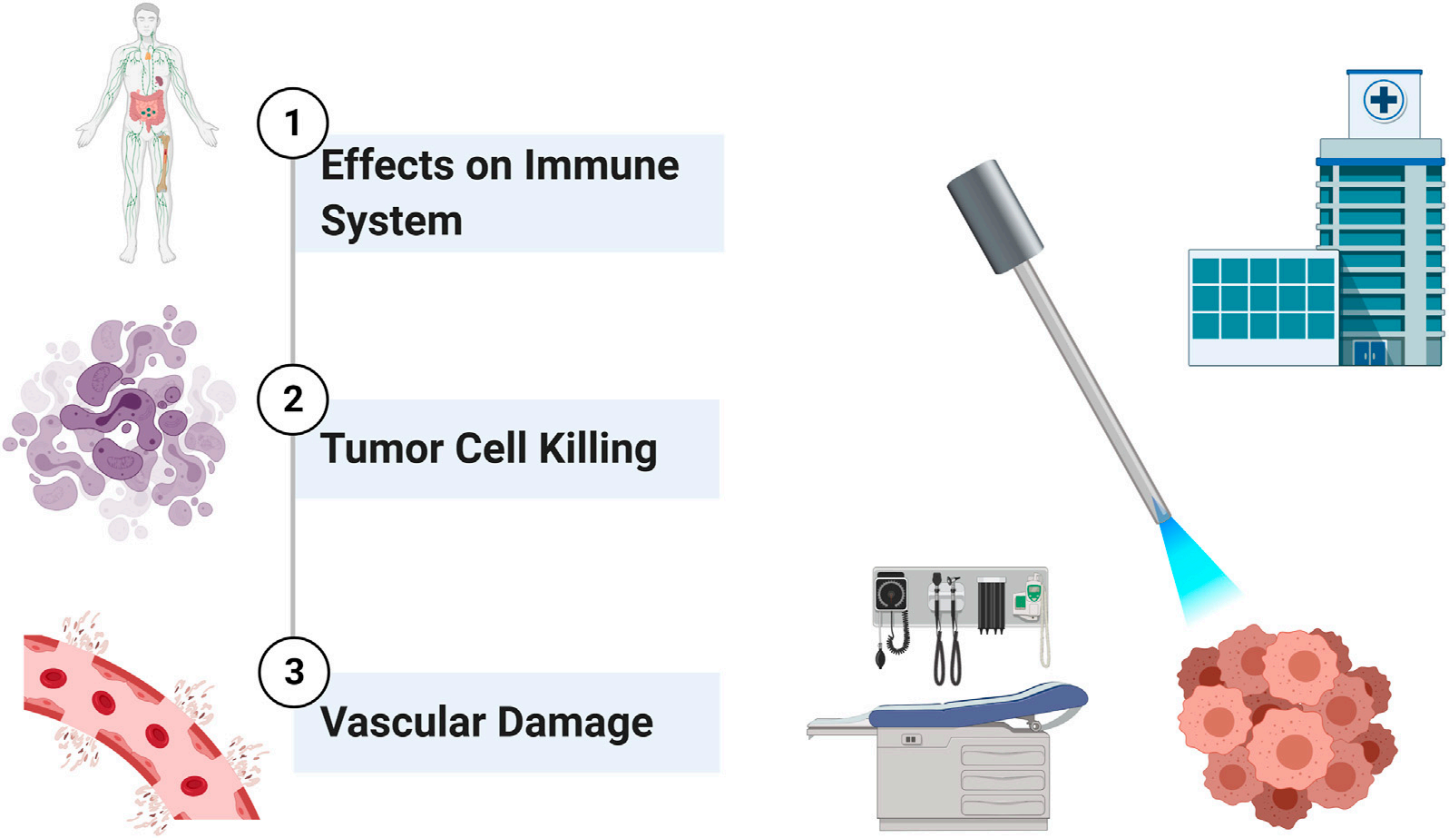
Basic Mechanisms of Action of PDT on Tumors. PDT can exert a plethora of actions such as inducing immune responses, direct killing of tumor cells, and damaging vascular structures.

PDT is an approach which utilizes specific agents that act as photosensitizers (Moore et al., 2009). Such chemicals are in inactive state until they are exposed to specific light. Their activity is also strictly dependent on the presence of oxygen. The photosensitizer (PS), which is activated in the presence of light, generates ROS. ROS, in turn, are responsible for the effector functions such as killing of tumor cells (Castano et al., 2006b). The excitation of the PS with light results in the move of an electron to the first excited singlet state. The following intersystem crossing yields a triplet state. The triplet PS transfers energy to triplet oxygen, resulting in the generation of reactive singlet oxygen (^1^O_2_). ^1^O_2_ is capable of exerting a plethora of actions such as direct killing of cancer cells, damaging vascular structures as well as inducing immune responses (Figure 1). PDT can induce cancer cell death *via* apoptosis and necrosis (Oleinick et al., 2002). In addition, PDT may harm vascular structures of the tumor, rendering cancer cells hypoxic and deprived of vital nutrients (Krammer, 2001; Dolmans et al., 2002b). The current literature has shown that ROS have various biological effects (Castano et al., 2005a; Castano et al., 2005b). Although one of the most potent effects of PDT is direct killing of tumor cells, PDT induced immune responses also have a great potential to alter the overall effectiveness of the treatment (Castano et al., 2006b). The net effect of PDT may be either the stimulation or the suppression of the immune system (Korbelik, 1996; Canti et al., 2002; van Duijnhoven et al., 2003).

Due to convenience, the first clinical examples of therapeutic applications of PDT are targeted against superficial diseases, *e.g.* lupus vulgaris and skin tumors. At the beginning of 20th century, Von Tappeiner and Jodlbauer published a book about the clinical utilization of PDT against infectious diseases and cancer (von Tappeiner and Jodlbauer, 1907). The first examples of PDT were mostly based on excitation by visible light and topical application of the PS, such as eosin. The problem to deliver light to the site of cancer other than superficial ones was achieved *via* the utilization of optical fibers, which could reach interstitial tumor tissues. Such advancements paved the way for the use of PDT against various cancer types including those of the head and neck, pancreas, prostate etc. (Bown et al., 2002; Lou et al., 2004). It is clearly evident that PDT can be implicated in several non-cancer clinical conditions such as acne vulgaris (Gold, 2007). The PS can be administered *i.v.* or *p.o.* for the treatment of interstitial tumors. Then, it is activated by means of a specific wavelength of light at the desired tissue site. A laser can be used for this purpose. The light can be applied to the target tissue through optical fibers within transparent plastic needles (Moore et al., 2009). Specific types of lasers or light-emitting diodes (LEDs) can be utilized for PDT. The type and body localization of the tumor tissue determine the kind of light that will be used. In order to precisely target the tumor tissue, the guides are placed by the help of ultrasonography, usually under general anesthesia. There exists a period of time between the administration of the drug to a patient and the application of light. This period, which can vary from hours to days depending on the PS in use (*e.g.* pharmacokinetic properties), is called as drug-to-light interval. Most PDT applications do not require hospitalization and they are commonly performed in an outpatient setting.

The route of administration of the PS can vary according to the site of the tumor. For instance, topical application is used for cutaneous lesions, whereas *i.v.* administration is frequently utilized for deep-seated cancer tissues. In such applications, it is conceivable that the pharmacokinetic properties of the agent assume pivotal roles. Thus, such kinetic features in fact determine the time of excitation of the PS at the target tissue by light. As explained, *vide supra*, inactive PS that is distributed to many sites of the body can then be activated in a site-specific manner. The excited PS transforms into the triplet state, which can undergo two kinds of reactions (Henderson and Dougherty, 1992). The activated PS can directly interact with oxygen to generate ^1^O_2_ (type II reaction). On the other hand, it can also react with a substrate such as a molecule or the cell membrane, to form radicals; which may react with oxygen to generate oxygenated products (type I reaction) (Dolmans et al., 2003). Since the fundamental mechanisms of PDT mandate the presence of oxygen, it is reasonable to foresee that such mechanisms would not occur in the absence of oxygen in tissues, *i.e.* anoxia. In line with this, seminal *in vivo* studies demonstrated that experimental induction of hypoxia diminishes the effects of PDT (Gomer and Razum, 1984). Although both type I and II reactions usually take place at the same time, the predilection toward either of them is dependent on the PS, presence and levels of oxygen as well as the type and levels of the substrate. Since the resultant ^1^O_2_ has a very short half-life, most of the effects of PDT are limited to the site of photosensitization (Moan and Berg, 1991). Moan *et al.* reported that the half-life of ^1^O_2_ in the cells was 0.01–0.04 μs and the distance diffused by ^1^O_2_ was estimated to be 0.01–0.02 μm (Moan and Berg, 1991). When all parameters of the PDT process are taken into consideration, the effectiveness of PDT significantly depends on several different variables such as the dose, type, and cellular localization of the PS; the intensity, duration and wavelength of light; as well as the availability of oxygen at the target site (Dolmans et al., 2003).

### Effects of Photodynamic Therapy on Tumors

The destruction of tumors by PDT is a phenomenon known for about a century. PDT’s net effect of tumor elimination encompasses several distinct mechanisms (Dougherty et al., 1998; Oleinick et al., 2002). PDT may stimulate the host immune system against tumor cells (Castano et al., 2006b). While conventional anti-cancer treatment modalities such as radiotherapy and chemotherapy mostly cause immune suppression, PDT is capable of inducing inflammation, recruiting leukocytes to the target area, as well as facilitating the activation of anti-tumor T lymphocytes. PDT also decreases the tumor microvasculature; thus, resulting in deprivation of oxygen and nutrients in the tumor tissue (Krammer, 2001; Busch et al., 2002). Last but not least, PDT is able to kill tumor cells directly *via* induction of apoptosis or necrosis by ^1^O_2_. These mechanisms are most likely interrelated (Figure 1). All or some of those mechanisms prove to be important in different clinical settings depending on the type of tumor and the PS. Tumor tissues not only contain cancer cells (parenchyma), but also the stroma (Peng and Nesland, 2004). Tumor microenvironment consists of various entities such as immune cells, fibroblasts, vascular structures, as well as extracellular matrix. Most of the stromal elements function to facilitate tumor growth. Most of the elements of tumor *milieu* can be affected by PDT.

#### Effects on Immune System

**FIGURE 2:**
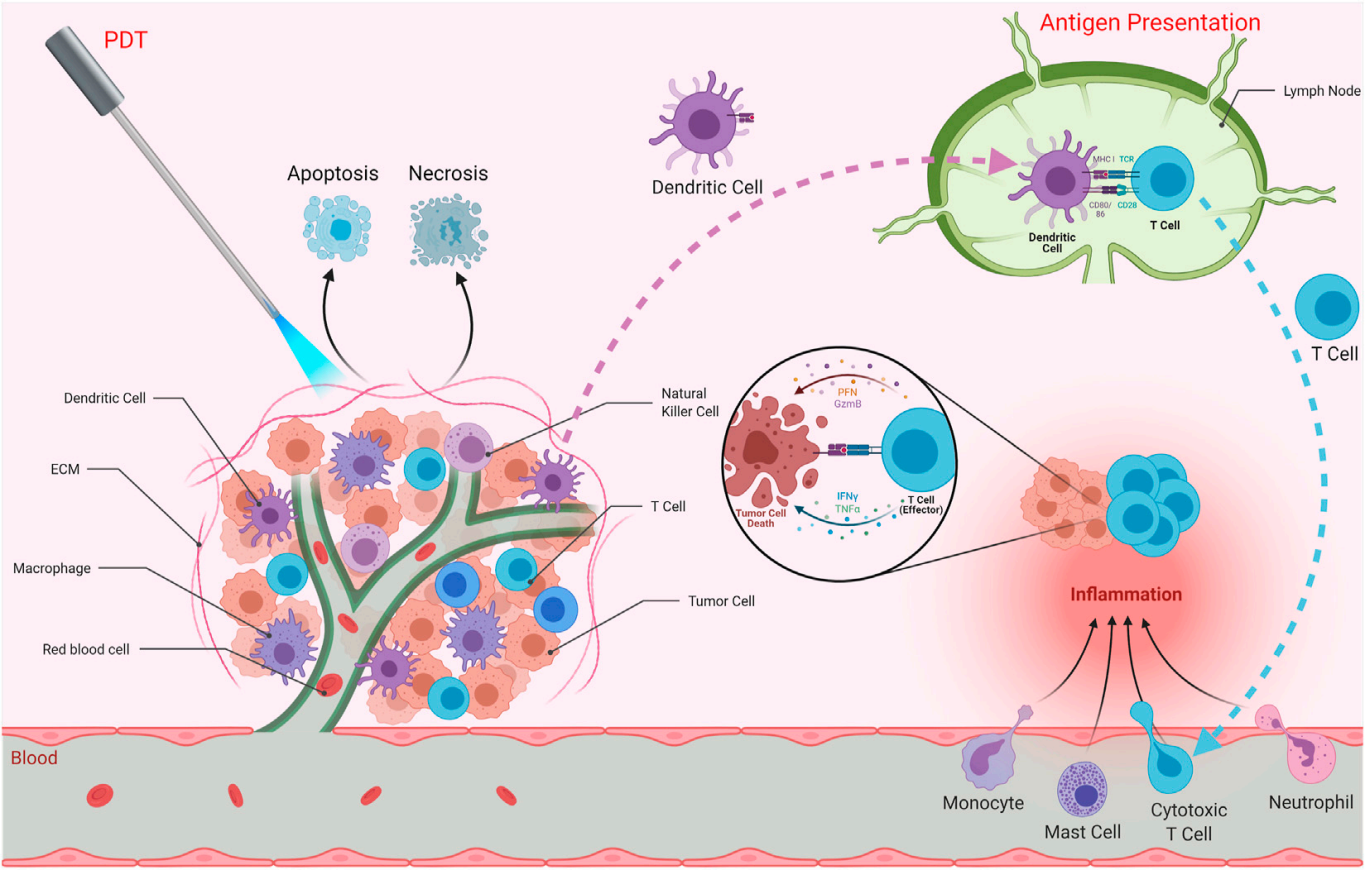
Effects of PDT on Immune System. PDT can affect immune responses and induce anti-tumor immunity as well as stimulating inflammation at the target tissue. PDT may result in apoptosis and/or necrosis of the tumor cells. It is also capable of inducing immunogenic cell death, which stimulates immune responses against dead cell antigens. The antigens are taken up by antigen presenting cells such as dendritic cells. These cells then travel to secondary lymphoid organs in order to present those antigens to T cells. Activated T cells as well as monocytes, mast cells and neutrophils are recruited to the tumor microenvironment, resulting in inflammation. Effector T cells are capable of eliminating tumor cells.

PDT has been shown to affect immune responses (Figure 2) (Korbelik, 1996; Canti et al., 2002; van Duijnhoven et al., 2003). Such effects can result in immunostimulation or immunosuppression. Conventional therapies such as chemotherapy and radiotherapy usually cause immune suppression. The tumor tissue that has been treated with PDT may provide important chemoattractant signals for immune cells. PDT can exert effects on both monocytes/macrophages and lymphocytes (Steubing et al., 1991; Jiang et al., 1999). Evans *et al.* demonstrated tumor necrosis factor (TNF) production by PDT treated macrophages, and proposed that this process might serve as a mechanism of PDT cytotoxicity *in vivo* (Evans et al., 1990). Gollnick *et al.* showed that PDT *in vivo* causes significant changes in the expressions of interleukin (IL)-6 and IL-10, but not TNF-α (Gollnick et al., 1997). PDT treated macrophages *in vivo* may display increased Fc receptor mediated ingestion activity (Yamamoto et al., 1991). Another study by Korbelik *et al.* reported an enhancement of macrophage mediated killing of tumor cells treated by PDT (Korbelik and Krosl, 1994b). Marshall *et al.* investigated the effects of photosensitizers on functional activities of macrophages and natural killer (NK) cells and reported that PDT may alter NK cell functions (Marshall et al., 1989).

PDT is capable of inducing acute inflammation at the target tissue (Castano et al., 2006b). Such an inflammatory response can be considered important for the activation of anti-tumor immunity (Gollnick et al., 2003). As a result of PDT, expressions of inflammatory cytokines [*e.g.* interleukin (IL)-6], chemokines and adhesion molecules [*e.g.* E-selectin and intracellular adhesion molecule (ICAM)-1] as well as infiltration of leukocytes (*e.g.* neutrophils) into the target tumor tissue were observed (Gollnick et al., 2003). In fact, suppression of tumor growth following PDT was proposed to be associated with the presence of neutrophils. Furthermore, it was proposed that neutrophils are pivotal for successful PDT *in vivo* (de Vree et al., 1996). Yom *et al.* suggested that a systemically mediated inflammatory response resulting from thoracic surgery followed by PDT is important. They found that IL-1β, IL-6, IL-8, and IL-10 levels were elevated after surgery and PDT (Yom et al., 2003). In another study, PDT was found to cause the release of prostaglandin E2 (PGE2) from mouse radiation-induced fibrosarcoma tumor cells and macrophages (Henderson and Donovan, 1989). Moreover, Henderson *et al.* found that endothelial cells could release prostaglandin F2α in response to PDT (Henderson et al., 1992). In summary, activation of inflammation is critical in terms of inducing potent immune responses.

The contribution of the immune system in tumor regression after PDT was investigated by several groups (Hendrzak-Henion et al., 1999). CD8^+^ T cells were shown to be required to prevent tumor regrowth after PDT. NK cells were also implicated in this response. PDT was able to induce protective anti-tumor immunity (Hendrzak-Henion et al., 1999). A study investigated the effects of photoactivated phthalocyanines on anti-tumor immune responses in immunosuppressed and normal mice having fibrosarcoma. The results suggested that PDT induced specific anti-tumor immunity was important (Canti et al., 1994). Another study investigated the effect of lymphoid cells in the response of murine EMT6 mammary sarcoma to PDT (Korbelik et al., 1996). PDT cured the lesions in BALB/c mice. On the other hand, PDT induced an initial ablation but no long-term cure for tumors in the immunodeficient strains (Korbelik et al., 1996). The results underlined the importance of lymphoid cells for preventing the recurrence of tumors after PDT and the role of PDT induced immune reaction (Korbelik et al., 1996). In another study, Korbelik and Dougherty demonstrated that cytotoxic T cells were the main immune effector cells responsible for the curative outcome of PDT, while helper T cells played a supportive role (Korbelik and Dougherty, 1999). They also reported NK cell activation in PDT treated sarcomas (Korbelik and Dougherty, 1999). Castano *et al.* demonstrated that benzoporphyrin derivative mediated PDT of poorly immunogenic murine sarcoma tumors led to initial tumor elimination, but all tumors recurred (Castano et al., 2006a). On the contrary, they observed complete cures and 100% mouse survival when they transduced the tumor cells with green fluorescent protein (GFP), which may act as a foreign antigen. Thus, PDT could then induce long term memory immune responses (Castano et al., 2006a). Such studies strongly underline the importance of the immune system in terms of effective clinical applications of PDT. Although various studies demonstrated augmented anti-tumor immunity after PDT; several studies also reported that PDT can cause immune suppression (Hunt and Levy, 1998). It was reported that there was no correlation between photosensitivity and immune suppression (Musser and Oseroff, 2001). In addition, the properties of the immune suppression are affected by the type and site of irradiation with light (Musser and Oseroff, 2001).

#### Tumor Cell Killing

PDT is able to eliminate tumor cells directly to some extent and such an elimination is not achieved through a single mechanism (Henderson et al., 1985). PDT is capable of reducing the number of clonogenic tumor cells. It was reported that up to approximately 72% reduction in the number of clonogenic cells could be achieved immediately after the completion of light irradiation of photosensitizer treated tumors (Chan et al., 1996). In addition, tumor cell killing seems to be a kinetic process, since the number of clonogenic cells further decreased as a function of time after PDT. PDT directly induces a mixture of apoptosis and necrosis on target tumor cells (Oleinick et al., 2002; Yoo and Ha, 2012). PDT is able to rapidly induce apoptosis (Kessel and Oleinick, 2010). In addition, PDT was also reported to be able to induce cancer cell death by autophagy, which is an important conserved cellular recycling mechanism (Kessel et al., 2006; Buytaert et al., 2007; Reiners et al., 2010; Agostinis et al., 2011; Yoo and Ha, 2012). Moreover, such mechanisms can occur concurrently, depending on the type and dose of the photosensitizer (Yoo and Ha, 2012). Agarwal *et al.* demonstrated that PDT could result in DNA degradation into fragments and induce apoptosis (Agarwal et al., 1991). The DNA fragmentation was affected by time and dose. They also reported the presence of chromatin condensation around the periphery of the nucleus as well as damage to cytoplasmic structures (Agarwal et al., 1991). PDT was reported to be a potent inducer of apoptosis in various conditions and several photosensitizers affect the mitochondria. There are numerous studies which investigate the mechanisms implicated in the PDT-mediated induction of apoptosis in cells as well as the role of signal transduction pathways on the response to PDT. PDT mediated apoptosis induction incorporates pathways coupled with Bcl-2 family members and caspases (Yoo and Ha, 2012). On the other hand, autophagy can take place in a Bax-independent manner, when the apoptotic pathway is unavailable or in parallel with apoptosis.

It has long been known that several cancer therapies including PDT target apoptotic pathways. PDT is able to induce different pathways of apoptosis (Buytaert et al., 2007; Ortel et al., 2009). Both the extrinsic (death receptor mediated) and the intrinsic (mitochondria mediated) pathways are associated with PDT mediated apoptosis. The types of the photosensitizer and the cancer cells affect the type of apoptotic pathway. PDT mostly triggers the intrinsic pathway and activates caspase-3 or caspase-7 (Buytaert et al., 2007; Ortel et al., 2009). Mitochondria assumes a pivotal role in the intrinsic pathway, which results in caspase dependent and independent apoptosis in PDT. It should also be noted that PDT primarily triggers caspase dependent apoptotic pathways (Yoo and Ha, 2012). Intracellular localization of the photosensitizer also affects the efficiency of apoptosis induction in PDT (Oleinick et al., 2002). In line with this, photosensitizers that localize to mitochondria are very potent inducers of apoptosis (Oleinick et al., 2002).

Several studies reported important findings concerning the apoptotic pathways that are triggered in response to PDT and key mechanisms associated with mitochondrial events. Various apoptosis mediators as well as signaling pathways have been uncovered in the setting of PDT (Moor, 2000; Agostinis et al., 2004; Castano et al., 2005b; Plaetzer et al., 2005). In addition to *in vitro* experiments, several studies demonstrated the *in vivo* apoptotic implications of photodynamic tumor therapy (Lilge et al., 2000; Chen et al., 2002a; Kaneko et al., 2004). One of the earlier events observed in PDT mediated apoptosis is the disruption of mitochondrial transmembrane potential. PDT may rapidly cause loss of mitochondrial membrane potential (Chaloupka et al., 1999; Kessel and Luo, 1999).

Kowaltowski *et al.* proposed that disruption of mitochondrial membrane potential in PDT might result from the effects of the PDT agent on inner membrane permeability to protons (Kowaltowski et al., 1999). Several studies suggested that photosensitizers that target the mitochondria might induce effective apoptosis in PDT. Mechanisms including cytochrome c release as well as activation of caspases such as caspase-9 and caspase-3 were reported to be associated with PDT (Granville et al., 2000; Xue et al., 2001b). In line with such findings, the role of caspase-3 in PDT mediated apoptosis has been investigated in many studies with various photosensitizers (Granville et al., 1997; He et al., 1998; Assefa et al., 1999; Inanami et al., 1999; Chan et al., 2000; Renno et al., 2000; Gad et al., 2001). PDT has also been reported to alter several signaling pathways which have been implicated in responses to oxidative stress (Moor, 2000). In summary, it is evident that PDT is very potent in triggering apoptosis (Oleinick and Evans, 1998). Nevertheless, PDT currently is not completely successful in eradicating tumors in the clinical setting. Several explanations have been proposed for the current state. It was suggested that the efficiency of PDT mediated killing of tumor cells decreases with increasing distance of the cells from the vascular supply and heterogeneous distribution of the photosensitizer in the tumor tissue may result in critical implications (Korbelik and Krosl, 1994a). Moreover, the availability and presence of oxygen in the target tumor tissue also affects the efficiency of PDT. This topic will be discussed in more detail. Many tumor tissues display hypoxia. In addition, PDT consumes oxygen during photodynamic processes. Last but not least, PDT is also capable of damaging vascular structures; causing oxygen deprivation. It has been found that PDT results in acute decreases in tissue oxygen levels (Tromberg et al., 1990; Pogue et al., 2001). Pogue *et al.* reported that the change in partial oxygen pressure in hypoxic tissue regions demonstrated acute loss after treatment, while the regions with higher partial oxygen pressure was heterogeneous, and some areas maintained their partial oxygen pressure value after the treatment (Pogue et al., 2001). In contrast, another study reported that tumor tissue partial oxygen pressure increased after the light application was finalized in a setting where the photosensitizer was injected 3 h before the application of light (Pogue et al., 2003). In fact, tissue hypoxia may limit the clinical effectiveness of PDT. Fractionation of the irradiation process of PDT may allow for the replenishing of oxygen in the tissue (Messmann et al., 1995; Pogue and Hasan, 1997; Turan et al., 2016).

#### Vascular Damage

PDT can damage the vascular structures of the tumor tissue. Henderson *et al.* demonstrated that *in vivo* treatment of tumors with PDT decreased the number of clonogenic tumor cells, *via* the damage to the tumor circulation (Henderson et al., 1985). The decrease in tumor clonogenicity 4 h after PDT closely resembled that of tumor deprived of oxygen for the identical period of time, suggesting that one of the important factors that contribute to tumor destruction might be the damage to the tumor vasculature and the consequences of treatment-induced alterations in tumor physiology. It is evident that the tumor cells heavily depend on nutrients and oxygen supplied by the vasculature. As such, many tumor cells were reported to secrete pro-angiogenic factors such as vascular endothelial growth factor (VEGF) (Bergers and Benjamin, 2003; De Palma et al., 2017). Therefore, cutting the supply lines of tumor *via* PDT mediated vascular shut down is a reasonable approach, which in turn results in hypoxia (Busch et al., 2002). Numerous studies have revealed that PDT bears the potential to significantly damage tumor blood vessels (Fingar et al., 1999; Dolmans et al., 2002b). In addition, several photosensitizers were demonstrated to decrease blood vessels and cause thrombosis (Star et al., 1986; Fingar et al., 1997; Fingar et al., 1999).

#### Signaling Alterations

**FIGURE 3:**
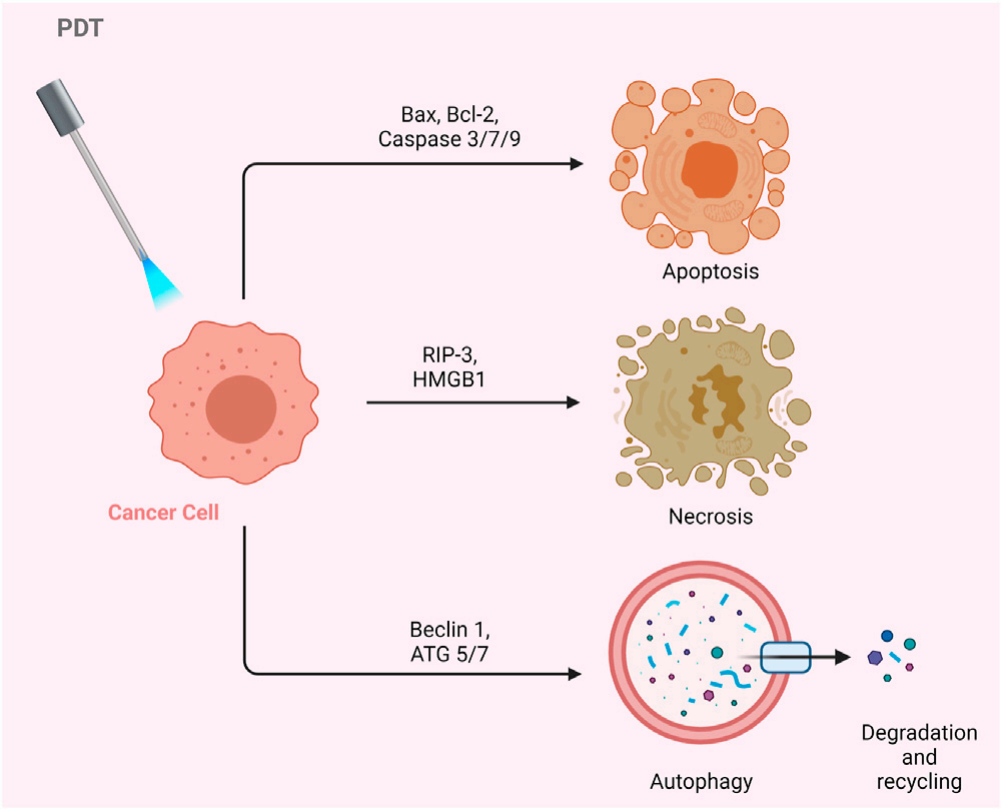
Effects of PDT on Cancer Cells. PDT may result in apoptosis, necrosis, or autophagy.

The biological activity of PS in the cell may vary depending on its type, localization in the cell, dose, as well as route of administration. Such differences may result in alterations in the regulation of cellular signaling mechanisms. Therefore, the cell can respond to PDT with several biological responses such as apoptosis, necrosis, or autophagy (Figure 3) (Chilakamarthi and Giribabu, 2017; Kwiatkowski et al., 2018).

**FIGURE 4:**
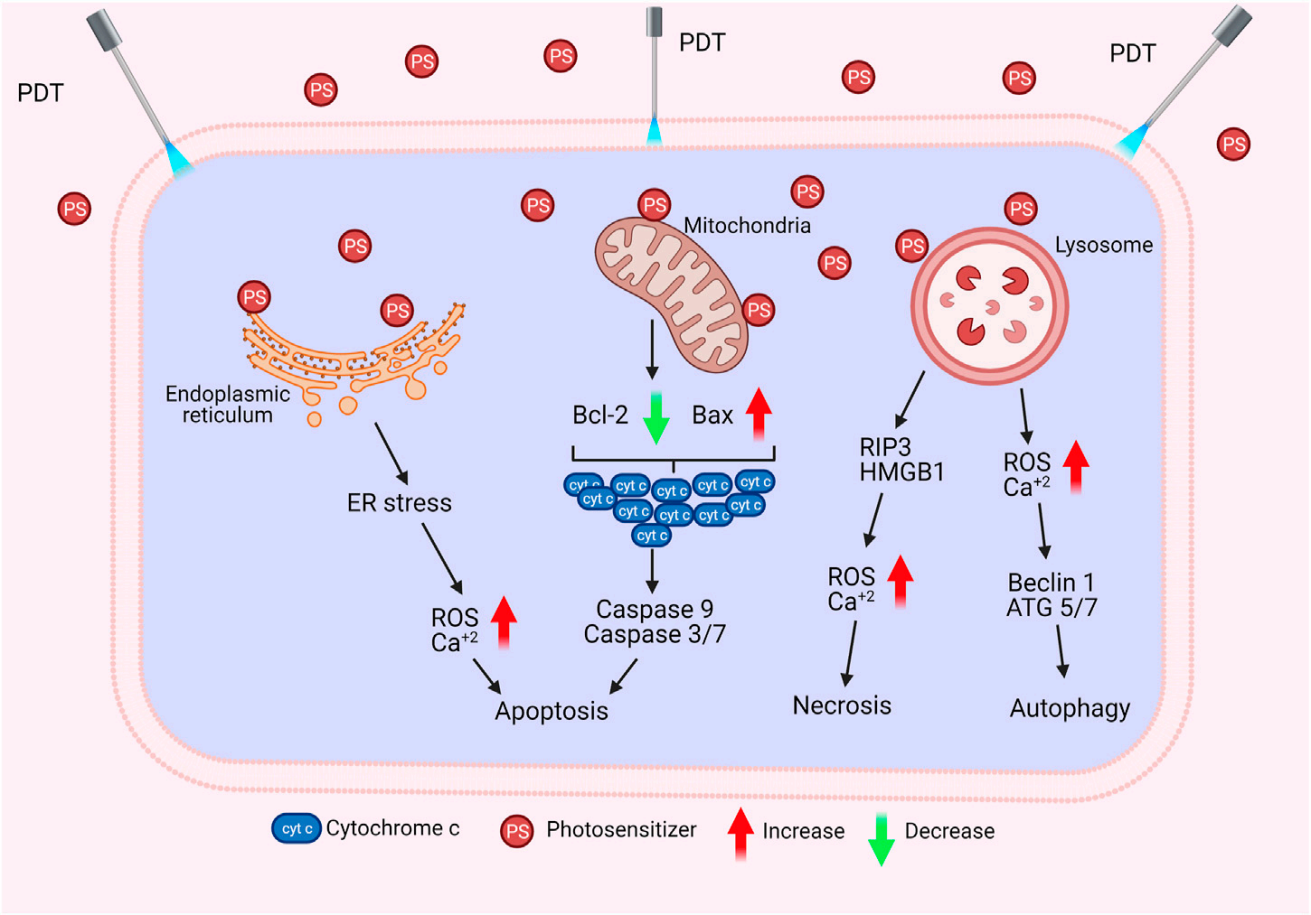
Cell Death by PDT. PDT may induce apoptosis, necrosis, or autophagy *via* different mechanisms.

Apoptosis is the programmed death of the cell. Indeed, PDT-mediated cellular damage can be achieved by crucial mechanisms of apoptosis, which are activated by the induction of pro-apoptotic proteins. The Bax/Bcl-2 ratio determines the fate of cells in terms of PDT-mediated apoptosis. When the pro-apoptotic proteins such as Bax are upregulated and the anti-apoptotic proteins such as Bcl-2 are downregulated, caspases that regulate apoptosis in the cell become activated (Figure 4). PDT may increase the levels of ROS in the cells and can also alter the concentration of Ca^2+^. It is known that subtle changes in Ca^2+^ concentration is able to regulate and trigger apoptosis. The p38-MAPK (mitogen-activated protein kinases) repair pathway is activated in normal cells under conditions of stress, such as genotoxic DNA damage, UV irradiation and hyperosmolarity. On the other hand, cancer cells use this signaling pathway as a *cat’s-paw* to prevent apoptosis and provide adaptive advantages such as proliferation, migration and invasion (Koul et al., 2013; Martinez-Limon et al., 2020). In a study conducted with different cell lines such as A431 (epidermoid carcinoma), HaCaT (immortalized keratinocytes), L929 (murine fibroblast) and HeLa (cervix adenocarcinoma), it was found that p38/MAPK signaling pathway was activated in cells as a result of hypericin-based PDT. It was demonstrated that activation of p38/MAPK signaling pathway could protect the cells from apoptosis (Assefa et al., 1999).

Necrosis is the premature death of the cell due to chemical or physical damage. Such a cellular death is usually considered as a violent and quick degeneration (Mroz et al., 2011). Necrosis can be characterized by swelling of the cytoplasm, organelle destruction as well as plasma membrane disruption. These changes may cause the release of intracellular contents and inflammation (Danial and Korsmeyer, 2004). It can be proposed that high dose PDT (*e.g.* high PS concentration and/or high light fluence) can result in cell death by necrosis. On the contrary, lower doses of PDT may have a predilection for apoptotic cell death (Mroz et al., 2011). Nagata *et al.* reported that PDT that caused less than 70% cytotoxicity resulted in mainly apoptosis; whereas, most of the cells appeared necrotic with doses that induced 99% cytotoxicity (Nagata et al., 2003). In another study, Dahle *et al.* reported that the mode of cell death induced by PDT depended on cell density (Dahle et al., 1999). They utilized meso-tetra (4-sulfonatophenyl) porphine as a PS. The apoptotic fraction was found to be higher for cells in confluent monolayers than those growing in microcolonies (Dahle et al., 1999).

Glioblastoma may respond to PDT with ALA, mainly by activating a necrotic cell death (Coupienne et al., 2011). Receptor-interacting protein 3 (RIP3) was suggested to be important in terms of this caspase independent form of programmed cell death. Coupienne *et al.* investigated the necrotic mechanism induced by ALA mediated PDT in human glioblastoma cells and demonstrated that PDT-induced necrosis is dependent on RIP3. They also proposed that PDT mediated ^1^O_2_ generation is responsible from RIP3 dependent activation of necrotic pathway (Coupienne et al., 2011). In addition, PDT was implicated in the formation of a pro-necrotic complex containing RIP3 and RIP1 but lacking caspase-8 and FADD (Coupienne et al., 2011).

Autophagy (self-eating) is an essential cellular mechanism, which is implicated in removal of unnecessary or dysfunctional components. Autophagy allows for the recycling of cellular components. In cancer cells, autophagy may contribute to cell growth. It has also been shown that photodamage may cause autophagy. As PDT augments the levels of ROS, cellular stress increases, and Beclin one protein induces the autophagic mechanism. PDT has been reported to be capable of inducing autophagy (Kessel and Reiners, 2007; Francois et al., 2011). PDT was proposed to induce autophagy by inactivating negative regulators of autophagy (*e.g.* mTOR, Bcl-2) rather than activating autophagic proteins (*e.g.* Atg7, Beclin 1, Atg5) (Xue et al., 2001a; Xue et al., 2003; Weyergang et al., 2009).

Several studies demonstrated that autophagy may show tumor suppressing or promoting effects depending on the type of PS used in the PDT application as well as the cell type and light flounce. A recent study with HCT116 and SW480 colorectal carcinoma cell lines proposed that PDT with *meta*-tetrahydroxyphenylchlorin (*m*-THPC) and verteporfin (VP) activated the ROS/JNK signaling pathway in cells. In addition, PDT induced autophagy mediated cell death through activation of the ROS/JNK signaling pathway (Song et al., 2020).

### Photodynamic Therapy for Non-Malignant Diseases

Clinical trials have adequately proved PDT’s effectiveness in treating various tumors. In addition to oncological conditions, current studies show the efficacy of PDT in the treatment of numerous non-oncological diseases. It was stated that PDT could be used in the treatment of various illnesses (Dougherty, 2002; Karrer and Szeimies, 2007). For instance, PDT is used to treat multiple dermatological and infectious diseases such as psoriasis vulgaris, Darier’s disease (DAR), cutaneous sarcoidosis (granulomatous disease), lichen planus, acne vulgaris, acne inversa, rosacea, sebaceous hyperplasia, verrucae vulgaris, cutaneous leishmaniasis, condyloma acuminatum (genital warts), and circumscribed scleroderma (Karrer and Szeimies, 2007; Babilas et al., 2010; Kharkwal et al., 2011; Queiros et al., 2020). In addition, PDT is used as a treatment method for dental ailments such as periodontitis, cardiovascular diseases such as atherosclerosis, esophageal varices, and neurological disorders such as Alzheimer’s disease and prion diseases (Meisel and Kocher, 2005; Al Habashneh et al., 2015; Yoo et al., 2021). Ophthalmologic conditions such as age-related macular degeneration (AMD), choroidal neovascularization (CNV, pathological myopia), gastrointestinal diseases such as Crohn’s disease, and musculoskeletal disorders such as rheumatoid arthritis and synovitis might be treated with PDT (Rivellese and Baumal, 2000; Yoo et al., 2021). In addition, it has been proposed that PDT can be an effective method in treating respiratory diseases such as pneumonia and COVID-19 (Yoo et al., 2021). While the aim of PDT in cancer is to cause cell damage, the main goal of PDT in the treatment of non-neoplastic diseases is the modulation of cellular function. For this reason, PDT is frequently modified for non-neoplastic diseases in terms of the treatment protocol. For instance, low dose PDT can be used to treat inflammatory skin diseases, in contrast to high dose PDT applications utilized for cancer treatment. Although there exist no standardized PDT protocols for non-neoplastic diseases yet, current studies demonstrate that PDT shows promising results (Karrer and Szeimies, 2007).

## Current Limitations of Photodynamic Therapy Utilization in Cancer

### Light

**FIGURE 5:**
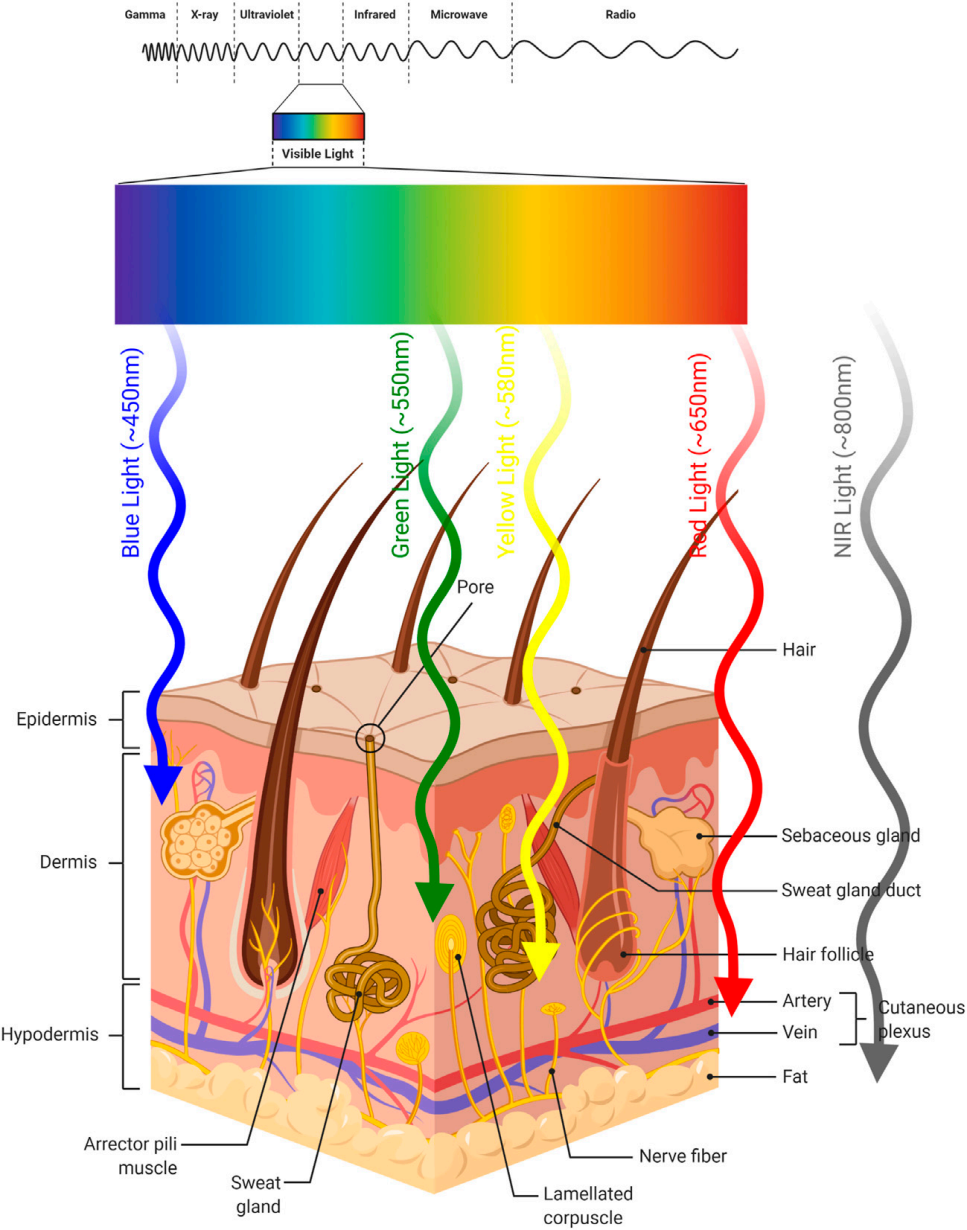
Light Penetration into Skin. Approximate penetration depths of light into skin according to its wavelength are illustrated.

**FIGURE 6:**
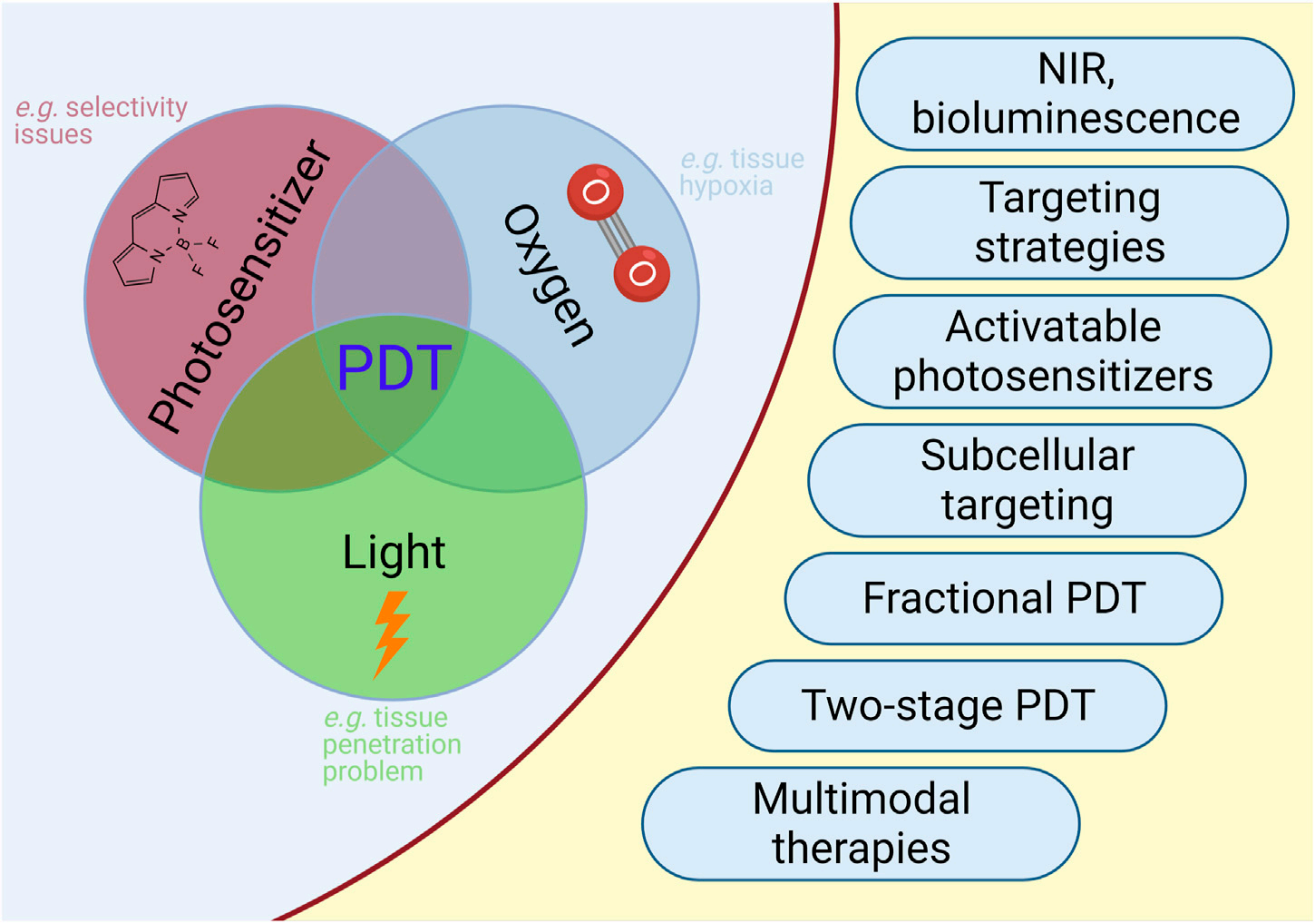
PDT in Cancer. Selected limitations and approaches to improve outcome of PDT in cancer are illustrated.

PDT is a treatment modality which consists of multiple components. PDT is a light-induced therapy and the therapeutic action of PDT is based on the properties of the light that is used to excite the particular chromophore. The requirement for excitation by light represents both an advantage and a disadvantage. The PS will not be active and generate cytotoxic singlet oxygen, unless it is excited. This brings an inherent selectivity to the procedure, since one can choose the timing and the area of irradiation. However, the light cannot penetrate (Stolik et al., 2000) beyond a few millimeters of tissue (Figure 5), limiting the therapeutic potential to superficial tumors (Figure 6).

The penetration of light depends on optical properties of the tissue and the wavelength of used light. There exists a heterogeneity between tissues and even within a tissue. These inhomogeneity sites (*e.g.* nuclei, membranes, etc.) cause light scattering, reflecting, transmitting or absorption (Mourant et al., 2000; Frangioni, 2003; van Straten et al., 2017). Moreover, water absorbs light at longer wavelengths, which affects light penetration depth into the tissue. In addition, hemoglobin and melanin, which are endogenous dyes, absorb light at shorter wavelengths. Therefore, they influence light penetration (Carroll and Humphreys, 2006). These results demonstrated that the light of spectral range (so-called “phototherapeutic window”) is important for the PDT. The “phototherapeutic window” is between 600 and 1,300 nm (Kim and Darafsheh, 2020). On the other hand, the light within the range of wavelengths between 620 and 850 nm has the most penetrating capability to achieve the maximum skin permeability. At above 850 nm, the light does not provide sufficient energy required to activate the PS, as a result PS cannot generate sufficient energy transfer to its triplet state to produce singlet oxygen. In conclusion, light within the 620–850 nm spectrum range achieves the optimum tissue penetration and PDT applications. The effective devices used for the delivery of light are incandescent light and lasers (Brancaleon and Moseley, 2002). There are some unique characteristics of the light source used for PDT. Thus, a universal light source cannot cover all types of PDT applications. First of all, the decision of light to be used is determined by the type of disease (tissue type, location and size of tumor). Secondly, a light source should be suitable for the spectral characteristics of the PS (e.g. absorption spectrum).

The power and density of light to be delivered are also important characteristics of the source. Coherent light sources (argon and argon-pumped lasers, solid-state lasers, metal vapor-pumped dye lasers, optical parametric oscillators lasers) and non-coherent light sources (fluorescent lamps, halogen lamps, metal halide lamps, xenon arc lamps, phosphor-coated sodium lamps) can be used for PDT (Gibson and Kernohan, 1993). For superficial lesions (*e.g.* skin, oral cavity), non-coherent light sources may be used, because they are cost-effective and commercially available, compared to a laser. Moreover, they can be used for various PSs, because of their broad emission range (Brancaleon and Moseley, 2002). On the other hand, lasers are the most widely utilized devices for the clinical applications of PDT. They provide a monochromatic, coherent light with very high-power output. Hence, they can reduce the time necessary for the application of PDT. In addition, they can be combined with optical fibers. This combination has proved to be useful in terms of illuminating deep-seated tumors (Brancaleon and Moseley, 2002).

Several recent studies have aimed at optimizing the light source for PDT. LED—based PDT is one of such approaches. The use of LED source provides practical advantages in PDT. For example, LED source is cheaper and easy to design and the field of irradiation is larger (Pariser et al., 2008; Hempstead et al., 2015; Erkiert-Polguj et al., 2016). Narrow emission band can be selected precisely to maximize the PS efficacy compared to the use of daylight (DL). Daylight can also be used as a light source in PDT (Cantisani et al., 2015). It is known that daylight cannot penetrate in deep tissues and its emission spectrum contains a large proportion of blue light, so this therapy is effective for superficial treatments such as non-melanoma skin cancer and actinic keratosis (Lacour et al., 2015; Assikar et al., 2020). The application of DL—PDT is safe, well tolerated and nearly painless as well as mostly nonsurgical. Moreover, light dose and fluence rate are other crucial aspects of PDT. By applying varying irradiation methods with the same light source, we can obtain different results in PDT. Low fluence rate was shown to promote tumor control; whereas, high fluence rate depleted the oxygen levels in tumor site in a very short time. Thus, depletion of oxygen may limit the efficiency of PDT. Such results demonstrated that *in situ* light dosimetry was crucial in terms of achieving optimum outcome (Robinson et al., 1998; Henderson et al., 2006). In another study by Robinson *et al.*, light fractionation extensively enhances the efficacy of ALA based PDT. The results showed that light fractionation compared to a single illumination significantly increased visual skin damage response in mouse skin (de Bruijn et al., 2016). In conclusion, the subject of light requires further improvements and new developments, in order to improve overall PDT efficacy and widen therapeutic applications (Chen et al., 2002b; Guo et al., 2015; Mordon et al., 2015).

### Oxygen

The therapeutic efficacy of PDT depends on the concentration of molecular oxygen in tumor tissues. On the other hand, it is well known that tumor tissues are deprived of oxygen as a result of their rapid growth and insufficient vasculature as well as increased oxygen diffusion distances (Vaupel et al., 2001). Hypoxic condition, which demonstrates oxygen concentrations of typically less than 1 μM, is a critical problem for PDT; given the fact that oxygen is a vital component of this therapy (Ceradini et al., 2004). In fact, it was observed that photosensitization itself depletes cellular oxygen levels quickly in both tumor models and clinical application of PDT. Thus, the light dose has to be carefully adjusted and the light should be introduced in pulses (fractionated), preferentially (Xiao et al., 2007). For this reason, PDT is considered a self-limiting modality, which causes its own inhibition. Hypoxic zones of solid tumors are highly resistant to certain chemotherapies and radiotherapies (Fuchs and Thiele, 1998; Rousseau et al., 2021). According to recent studies, drugs such as carboplatin, cyclophosphamide and doxorubicin show hypoxia induced resistance to chemotherapy (Wouters et al., 2007). Similarly, the effectiveness of PDT is significantly reduced when the major vessels of the tumor tissue are shut down (Fingar et al., 1992). As a result, hypoxic tissues alter the optimal drug dose required for PDT (Harrison et al., 2002).

In order to overcome the problems associated with low concentration of oxygen in solid tumors, hyperbaric oxygen (HBO_2_) therapy was combined with PDT. The results showed that HBO_2_ enhanced tumor oxygenation. Thus, a significant improvement in PDT efficacy could be obtained when applied during hyper-oxygenation (Al-Waili et al., 2005). In another study by Hetzel *et al.*, Photofrin-PDT was applied in combination with hyperoxygenation in order to overcome hypoxia. Mice with transplanted mammary carcinoma tumors were kept under three atp (atmospheric pressure) hyperbaric oxygen during the irradiation phase of PDT. The results showed that PDT can be more potent in controlling hypoxic tumors when combined with hyperoxygenation, (Chen et al., 2007b; Huang et al., 2007). In conclusion, molecular oxygen was indeed a fundamental element for PDT-induced cytotoxicity. The light fluence rate is related with photochemical oxygen depletion during the generation of cytotoxic singlet oxygen by PDT (Robinson et al., 1998). In another study by Snyder *et al.*, fluence rate was shown to affect the PDT tumor response in the Colon 26 tumor model. Lower fluence light promoted tumor control. Higher fluence light, on the other hand, significantly decreased oxygen concentration in the tumor tissue, swiftly (Robinson et al., 1998; Henderson et al., 2006). Furthermore, tumor oxygenation levels were investigated both before and after the application of PDT. The results showed that tumor oxygenation at the time of PDT has a profound effect on post-treatment tumor oxygenation. Such effects might be because of PDT cytotoxicity and PDT damage to tumor vasculature (Star et al., 1986; Chen et al., 1996). The therapeutic action of PDT depends on the efficient generation of singlet oxygen from the triplet oxygen (^3^O_2_); therefore, tissue oxygenation seems to be extremely important for the efficacy of PDT (Lee See et al., 1984; Moan and Sommer, 1985). In conclusion, the choice of optimal combinations of PS dose, light sources, and treatment parameters are very important in order to achieve successful results in PDT (Henderson et al., 2006).

### Photosensitizer (PS)

PS is another important component associated with PDT activity. PSs are generally accumulated in malignant areas. Subsequently, it is activated by a light source of specific intensity and wavelength. The illuminated PS interacts with oxygen and performs a photodynamic reaction (PDR). The anti-tumor effects that occur *via* PDR are directly related with PS activity. In this context; purity, pharmacokinetic properties, amphiphilicity and dosimetry of PS are important parameters in terms of treatment efficiency. Targeting different subcellular compartments of the target cell by using different molecular carriers of PS is another factor that may increase PDT efficiency. The fluorescence features of PSs can also be used for theranostic purposes in addition to PDT (Allison and Sibata, 2010; Allison and Moghissi, 2013; Erbas-Cakmak and Akkaya, 2013; Allison, 2014; Turkoglu et al., 2020). For instance, theranostic approaches can integrate hypoxia imaging and tumor therapy (Fan et al., 2019; Zheng et al., 2020).

Some of the features that should be found in an ideal PS are low dark toxicity, easy handling, high activation capacity and singlet oxygen yield. Several PSs have demonstrated low solubility and aggregation in water, which may cause them to be photodynamically inactive in aqueous solutions. This issue can hinder *in vivo* utilization of such PSs (Dumoulin et al., 2010; Li et al., 2015). Most studies focus on the absorption and emission properties of PSs in DMSO solutions (Sekhosana and Nyokong, 2014). An optimal PS should demonstrate water solubility and high singlet oxygen quantum yield in aqueous solution. Insoluble PSs were previously incorporated in liposomes, nanoparticles or emulsions in order to be able to use them in aqueous solutions (Jacques and Braun, 1981; Ricci-Junior and Marchetti, 2006; Kuruppuarachchi et al., 2011; Rossetti et al., 2011). Hydrophilic substituents such as ionic substitutions can also be attached to PSs in order to enhance water solubility and singlet oxygen generation in aqueous solutions (Dubuc et al., 2008). In addition, nonionic water soluble PSs could be synthesized through modification with functional groups (*e.g.* carbohydrate and polyhydroxylate) (Zorlu et al., 2009).

## Novel Approaches to Improve Outcome

### Overcoming Problems Related With Light and Drug Dose in Photodynamic Therapy

The irradiation with light can be used as an external “ON-OFF” switch controlling PDT for tumor tissues *in vivo*. Light penetration severely limits the potential of PDT in deep tissues. It is very important to overcome the short penetration depth of light in PDT. Therefore, several studies aimed to design different light sources, devices and targeted delivery protocols in PDT. The excitation of PS can be achieved by using NIR light, x-ray radiation, *via* bioluminescence approaches, radioluminescent nanoparticles and quantum dots. The aim of these approaches is to increase PDT efficacy in deep-seated tumors (Bakalova et al., 2004; Juzenas et al., 2008; Burgess, 2012; Turan et al., 2014; Kolemen et al., 2016; Mallidi et al., 2016; Ozdemir et al., 2017). The sensitizer dye is preferentially designed to have strong absorption bands in the red to NIR region of the visible spectrum. This spectrum region minimizes the scattering of light due to tissue heterogeneity, reaches the penetration depths exceeding several mms and enhances the signal to noise ratio due to the low background emission in NIR (Oleinick and Evans, 1998). Designing of two-photon excitation sensitive PSs aim to enhance the treatment penetration depth with NIR light excitation (Bolze et al., 2017). These PSs can be combined with nanoparticles to increase the therapeutic efficiency (Chen et al., 2014). In a study by Akkaya *et al.*, a NIR absorbing BODIPY derivative sensitizer was synthesized. The BODIPY-based PS produced cytotoxic singlet oxygen in the presence of high intracellular glutathione (GSH) concentrations within cancer cells (Turan et al., 2014). Besides NIR light, X-ray radiation is also an effective candidate as an indirect excitation energy source for the therapeutic application of PDT for deep-seated tumors (Fan et al., 2016; Ni et al., 2018). However, it is clear that there exists an energy mismatch between the photosensitizer (singlet–triplet energy gap of eV) and the therapeutic X-ray (photon energy of keV–MeV). As a result, this design requires the use of induced Čerenkov radiation or radioluminescence of nanoparticles (scintillation) that produce light upon X-ray excitation, which can activate nearby PSs. Alternative routes can be utilized in order to overcome depth limitations in addition to the use of external excitation sources. In addition to these studies, the auto-PDT strategy, which aims to increase PDT efficiency without requiring the presence of an external light source, has also been utilized (Blum et al., 2020). A self-illuminating nanoparticle was designed as a PDT agent which can be excited by enzyme-mediated bioluminescence approaches (Fan et al., 2016; Ozdemir et al., 2017). Persistent luminescence is another alternative method which may provide an internal light source to generate cytotoxic singlet oxygen in PDT. Nanoparticles that emit NIR light with long luminescence lifetimes can excite PSs for a long duration. Hence, PDT may then be emancipated from the requirement for external light (Abdurahman et al., 2016; Fan et al., 2017).

Adjusting the dose of photosensitizing agent in the clinical setting is one of the major approaches in terms of overcoming the limitations of PDT. Administration of fractional drug doses provides a new strategy to optimize PDT therapy. In a study by Dolmans *et al.*, the fractioned photosensitizer (MV6401) doses show more efficacy in inducing tumor delay compared to the same total dose given as a single treatment in an orthotropic breast tumor model (Dolmans et al., 2002a). The metronomic photodynamic therapy (mPDT) represents a strategy in which both the light and photosensitizer are delivered consistently at low rates over several hours. The mPDT method was investigated for the treatment of preclinical rat models of brain tumors. Laser diode or LED was used as the light source and ALA was administrated through the drinking water. The researchers proposed that the study supported the concept of developing ALA-mPDT *in vivo* as a treatment for brain tumors. (Bisland et al., 2004). In summary, mPDT may increase the selective tumor cell killing through apoptosis.

### Selectivity of Photodynamic Therapy

#### Bioengineering Approaches

Given the fact that most common side effect of PDT is light sensitivity and that PDT in fact augments immune responses against tumor cells (in contrast to chemotherapy/radiotherapy), it is frustrating to see that such a treatment is not widely used in treating cancer patients. PDT strategies and techniques should be improved in terms of clinical applications and ease of use in order to compete with other procedures such as surgery or radiotherapy. Even though PDT applications in malignant tissues (*e.g.* ophthalmological and cardiovascular diseases) were thoroughly explored with the advent of new generation of photosensitizers, further studies are still required (Rockson et al., 2000; Sivaprasad and Hykin, 2006). As a result, third generation PSs incorporated targeting and delivery moieties such as mAbs, liposomes as well as ligands which target specific receptors that are overexpressed (Chen et al., 2010). Furthermore, the required drug dose of PDT is relatively high in order to achieve therapeutic efficacy, because of the limited selectivity of the PDT agents. The light dose has to be carefully adjusted and the light can be applied preferably in pulses (Xiao et al., 2007). The applications of PDT in deep-seated tumors are limited due to light penetration problems (*vide supra*). Moreover, hypoxia and issues related with skin toxicity severely limit the potential of PDT. As a result, we will discuss the approaches to overcome these limitations below.

#### Targeting Strategies

There are two main strategies in terms of targeting the drugs to tumors; passive targeting and active targeting. As part of the passive targeting strategies, various nanocarrier agents have been used to promote their particular accumulation within tumors through the enhanced permeability and retention (EPR) effect (Zhang et al., 2020). The EPR effect is a common concept for solid tumors associated with their pathophysiological and anatomical differences from normal tissues (Matsumura and Maeda, 1986). The EPR effect is manifested by enhanced accumulation of macromolecules (*e.g.* liposomes, drugs, and NPs) in tumor tissues in contrast to normal tissues. The mechanism underlying this effect is attributed to the leaky vessels around tumors as well as dysfunctional lymphatic system (Perrault and Chan, 2010; Maeda, 2013). The blood vessels with such leaky pores can augment the transport of circulating nanoparticles into the tumor. On the other hand, the transportation in non-malignant tissues is hindered by the intact barrier of the vasculature. EPR effect has been proposed to be due to the rapid growth of tumor cells, which require surplus amounts of crucial nutrients; thus, inducing the dysregulated formation of blood vasculatures. The size of nanoparticles should be between 10 and 200 nm to achieve the passive targeting effect (Allison et al., 2008). The EPR effect is also dependent on other features of nanoparticles, such as the charge, the shape, circulation time in blood and hydrophilicity (Bertrand et al., 2014). Even though the EPR effect has been demonstrated to have promising results in preclinical *in vitro* studies, there still exist important issues that should be solved in order to translate this approach into the clinical setting (Fang et al., 2011). The passive targeting strategy may fail to achieve the desired favorable therapeutic effects in certain early stage tumors, because of the regular vasculature of such tissues. Furthermore, vessel leakiness and permeability may not be homogeneous in a tumor tissue. Thus, targeting *via* EPR might yield heterogeneous effects (Peer et al., 2007). Moreover, tumor microenvironment in human cancers and murine models show some important differences. Therefore, “EPR” and “nanomedicine” approaches need reconsideration for human tumors (Danhier, 2016).

On the other hand, the active targeting strategy more specifically and effectively targets the tumor tissue compared to the EPR effect alone. Active targeting strategy includes the use of high-affinity ligands (peptides, antibodies, nucleic acids, vitamins or carbohydrates) that can be attached to the nanoparticle surface. Thus, it will predominantly bind to overexpressed receptors (ligand-receptor interactions) at the target site (Lammers et al., 2012). Arginine-glycine-aspartate peptide and epidermal growth factor have been used for the active targeting of PDT agents (Fang et al., 2012; Conde et al., 2016). Proteins, especially transferrin and antibodies, can be also utilized as targeting agents (Kotagiri et al., 2015; Wang et al., 2016). Last but not least; carbohydrates (Yoon et al., 2012), vitamins (biotin and folic acid) (Li et al., 2017b; Poudel et al., 2021) and aptamers (Farokhzad et al., 2006) have been implicated in active targeting of PDT agents.

The combination of the phototoxic effects of hematoporphyrin and the targeting ability of monoclonal antibodies were used for anti-cancer treatment, which was called as “photoimmunotherapy” (PIT) by Levy *et al.* (Mew et al., 1983). Furthermore, photoimmunotherapy technique has been developed also by other groups (Oseroff et al., 1987). Oseroff *et al.* proposed an approach, antibody-targeted photolysis (ATPL), which utilized PSs attached to monoclonal antibodies (mAbs). These mAbs bind to cell surface antigens on malignant cells. The results showed that cell death was dependent on the doses of both light (630–670 nm) and mAb-PS (Oseroff et al., 1987). Moreover, several groups have recently investigated PIT strategies to increase targeted therapy efficacy (Zhang and Li, 2018; Wang M. et al., 2021).

Schmidt *et al.* published the first clinical application of antibody-targeted photoimmunotherapy in 1992 (Schmidt et al., 1992). They demonstrated the use of an antibody-targeted phthalocyanine induced PDT, which resulted in cancer cell killing in three patients with advanced ovarian carcinoma (FIGO III). In another study by Kobayashi *et al.*, a new model of molecular targeted photoimmunotherapy was developed by using a NIR phthalocyanine dye (IR700), combined to anti-EGFR mAb (ASP-1929) (Mitsunaga et al., 2011). Clinical phase III trial of ASP-1929 PIT treatment in combination with anti-PD1 therapy is still ongoing in patients with recurrent head and neck squamous cell carcinoma (NCT04305795).

In addition to passive and active targeting strategies, physical forces (*e.g.* electric or magnetic field) may be utilized in order to enhance PDT efficacy. Superparamagnetic nanoparticles (*e.g.* Fe_3_O_4_) are frequently used as nanocarriers for PDT. They can be combined with a photosensitizer, which has a strong optical absorption band in the NIR region, so that they can be targeted to tumor tissues as a result of external magnetic field application. NIR laser irradiation is then applied to initiate cancer cell death. Such approaches significantly increase the effectiveness of PDT (Cheng et al., 2012; Dou et al., 2015; Wang et al., 2017). As discussed *vide supra*, the PDT agents have been designed to accumulate in the extracellular matrix of tumor microenvironment or to selectively bind to the extracellular layer of the cancer cell membrane. In addition to these strategies, subcellular targeting of PSs can be used in order to enhance the therapeutic efficacy of PDT. Subcellular targeting approaches will be discussed in detail below.

#### Activatable Photosensitizers

Considering the fact that PSs are mostly in “ON” state, light sensitivity in healthy tissues due to sunlight is a common side effect of PDT. Therefore, patients undergoing PDT should avoid direct sunlight after PDT treatment until the PS is eliminated from the body, in order to prevent severe light sensitivity in healthy tissues. Possible side effects after PDT treatment include skin lesions and the ocular adverse events (Vrouenraets et al., 2003; Huang, 2005). In order to improve patient prognosis and enhance PDT response, activatable photosensitizers have been developed (Li et al., 2017c; Gharibi et al., 2019; Digby et al., 2020; Liu and Li, 2020; Sun et al., 2020; Yuan et al., 2020; Zheng et al., 2020). The activatable photosensitizer designs can be used as an “ON/OFF” switch controlling PDT for effective cytotoxic singlet oxygen generation in the tumor tissue *in vivo*. It remains in an “OFF” state (inactivated PS), unless activated by chemical/biochemical parameters into the “ON” state (activated PS) in the tumor region. Examples of such parameters are overexpressed enzymes/proteins (Yuan et al., 2014; Digby et al., 2020) and higher intracellular glutathione concentration in cancer cells (Cakmak et al., 2011; Turan et al., 2014; Erbas-Cakmak et al., 2015; Kolemen et al., 2015); hydrogen peroxide (Wang Y. et al., 2021); low pH of the environment (Tian et al., 2015); hypoxia (Ayan et al., 2020) and cathepsin B (Kim et al., 2014). The working principle of this switch generally involves cleavage of an intramolecular linker, which is connected to the PS as a quenching moiety. After activation (such as by enzymes, GSH), the PS becomes functional upon cleavage of the linker, resulting in the generation of singlet oxygen. In fact, “OFF-ON” switching of singlet oxygen generation according to cancer-related parameters has become a very crucial approach for researchers aiming to advance precision medicine (Erbas-Cakmak et al., 2013). Moreover, other designs may also utilize the “ON/OFF” switch controlling in PDT (Yurt et al., 2019). A 1:2 demultiplexer based on a Zn^2+^-terpyridine-Bodipy conjugate was reported to remain in an “ON” state, unless ^1^O_2_ generated by photosensitization triggered apoptotic response, which resulted in autonomous switching “OFF” of ^1^O_2_ generation (Turan et al., 2018). Another approach, which allows for a switchable strategy for photodynamic-immunotherapy, takes advantage of a switch that controls the ^1^O_2_ generation of self-assembled albumin nanotheranostics (Cheng et al., 2021). In another study, Yoon et al. reported a nanomaterial which self-assembles from phthalocyanine building blocks. They demonstrated that fluorescence and ROS generation may be activated based on a protein-induced partial disassembly mechanism (Li et al., 2017b). Moreover, heavy-atom-free PSs can also be utilized in terms of PDT. Replacing oxygen atoms in conventional naphthalimides with sulfur atoms was reported to cause important alterations in photophysical features. Sulfur substitution could enhance the intersystem crossing from the singlet excited state to the reactive triplet state. Such a molecular design, which has “OFF-ON” ROS generating properties depending on albumin as a targeted protein to disassemble intact 4-R substituted thionaphthalimides to the constituent monomers, has the potential to function even under hypoxic conditions (Nguyen et al., 2019).

### Subcellular Targeting in Photodynamic Therapy

The approaches related with the selectivity of PSs are aimed at increasing the selectivity of PDT to tumor tissues; therefore, augmenting the specificity of the treatment. In addition to tumor tissue targeting, PSs can also be modified so that they specifically target subcellular compartments or organelles. Such approaches have the potential to potentiate the effectiveness of PDT (Chen et al., 2019b; Li et al., 2020a). Targeting groups (*e.g.* triphenylphosphonium) are very important in PDT in order to direct the delivery of PS’s to the most critical subcellular organelle in terms of singlet oxygen mediated apoptosis. This approach can improve the efficacy and selectivity of PDT.

^1^O_2_ has long been known to have an extremely short lifetime, since it is highly reactive (Sharman et al., 2000). Even though ^1^O_2_ has a lifetime of approximately 3 µs in water, its lifetime in cells is estimated to be about 200 ns, because of its high reactivity with biological substrates (Krasnovsky, 1981; Egorov et al., 1989; Baker and Kanofsky, 1992). The quenching in water is facilitated by the electronic to vibrational energy conversion, which results in deactivation through interactions with the vibrational states of O–H bonds. Such a short lifetime of ^1^O_2_ results in a short diffusion range in cells, which was predicted to be approximately 45 nm (Moan and Boye, 1981). Given the fact that most human cells are between 10–100 µm in diameter, the site of generation of ^1^O_2_ determines the subcellular target on which it will mostly attack, as well as confining the photodamage to the areas where the PS is located (Kessel and Reiners, 2015). In fact, the sizes of most cellular organelles are much bigger in respect to such a short diffusion range, given many organelles are roughly about 1–5 µm (*e.g.* mitochondrion: ∼ 1–3 µm). Therefore, it seems clear that the subcellular location of the PS has a major impact on the effectiveness of PDT. As a result, several studies investigated the effects of utilizing organelle targeted PSs, which localize to nucleus, mitochondria or lysosomes (Karaman et al., 2019; Ucar et al., 2019). Indeed, such approaches might improve the outcome of PDT (Wan et al., 2020; Qiao et al., 2021). According to the literature, mitochondria-targeting groups (*e.g.* triphenylphosphonium) and nucleus-targeted peptides direct the agents to the most critical organelles, mitochondria and nucleus, respectively, in order to effectively initiate singlet oxygen mediated apoptosis (Morgan and Oseroff, 2001; Chen et al., 2019a).

#### Nucleus-Targeting

The nucleus is a crucial cellular organelle. Several anti-tumor agents target the DNA inside the nucleus. Such approaches are regarded to be effective in terms of tumor cell destruction. Vankayala *et al.* designed nucleus targeting gold nanoclusters to achieve NIR light activated PDT. They reported that the gold nanoclusters performed efficient nucleus-targeting PDT on tumor cells through photoinduced DNA damage (Vankayala et al., 2015). In another study, Chen *et al.* developed a nucleus delivery platform based on C_5_N_2_ nanoparticles and proposed that these nanoparticles might have a great potential in PDT (Chen et al., 2019a). Akhlynina *et al.* demonstrated that targeting chlorin e6 to the nucleus, presumably a hypersensitive site for oxygen species mediated damage, augmented the photosensitizing activity of chlorin e6, significantly reducing the EC_50_ (Akhlynina et al., 1997).

Nucleus-targeted PDT induce apoptosis *via* DNA damage. The singlet oxygen generation results in the inactivation of DNA repair enzymes as well as breaking DNA strands in nuclei (Ling et al., 2012). Based on these two important damage mechanisms, targeting the hypersensitive nuclei is considered as an important component of PDT (Devi et al., 2020; Wan et al., 2020). Shi *et al.* synthesized TAT and RGD (R: arginine, G: glycine, D: aspartic acid) peptides which are conjugated to mesoporous silica nanoparticles (MSNs). This PS was designed as an effective nuclear-targeted delivery strategy. The accumulation of PS inside nuclei can generate cytotoxic singlet oxygen upon irradiation. Thus, such a specific PDT with low side effects and high efficacy could be achieved *in vivo* (Pan et al., 2014). Due to the preparation and modification difficulties of such small-sized nanocarriers, nuclear targeting may also be achieved by using a different road map. Han *et al.* reported a simple and easy-to-fabricate delivery system. They designed an amphiphilic chimeric peptide (PAPP–DMA) which was used to realize sequential acidity-responsive tumor-targeted delivery of PS (Han et al., 2016). PAPP–DMA contained an alkylated PpIX, a PEG-linker and nuclear localization sequence (NLS) peptide (sequence PKKKRKV) modified with acidic liable 2,3-dimethylmaleic anhydride (DMA). Tumor acidic environment triggered charge reverse of PAPP-DMA NPs, resulting in accelerated cellular uptake of positively charged NPs. After the NPs specifically entered the tumor cells, NLS peptide achieved the intranuclear delivery of PS. *in vivo* and *in vitro* studies showed the anti-tumor efficacy of nucleus-targeted PDT.

The upconversion nanoparticle (UCN)-based nanoplatform was also used for nuclei-targeted PDT (Lucky et al., 2015; Chen et al., 2017). UCNs, which behave as transducers that convert NIR light to UV-VIS light, can be combined with PS (Lucky et al., 2015). UV-VIS light can then excite the combined PS. Titanium dioxide (TiO_2_) nanomaterial is commonly used for this platform, because of its nontoxicity and high photostability (Chen and Mao, 2007). Tang *et al.* combined a nano-UCNP@TiO_2_, molecule-PS (Ce6) and TAT (nuclear targeted peptides) in one platform. UCNP@TiO_2_-Ce6-TAT absorbed light at 980 nm and then converted that light into the wavelengths of 362 nm (absorbed by TiO_2_ shell) and 655 nm (absorbed by Ce6 molecules); so that multiple ROS were generated in nuclei by the help of TAT (Yu et al., 2016). *in vitro* and *in vivo* results confirmed the excellent therapeutic effects of nucleus-targeted PDT by UCN-based approach.

#### Cell Membrane-Targeting

The cell membrane (or plasma membrane) acts as a protective barrier, which is crucial for cellular integrity as well as intracellular metabolism and transportation of nutrients between extracellular and intracellular environment. Hence, cell-membrane-targeted PDT may prove to be very efficient, as it can attenuate the stability of the membrane, resulting in cell death (Wang et al., 2014). One of the advantages of cell-membrane-targeted PDT is that PSs do not need to cross the cell membrane. On the other hand, it is difficult to design a PS that stays anchored on cell membrane for long-term, due to cellular uptake and cellular endocytosis. In order to overcome this limitation, a new PS approach was proposed. This pH-driven membrane-anchoring PS (pHMAPS) design took advantage of pH low insertion peptide.

(pHLIP), which could insert across lipid bilayer at pH < 7 through conformational self-transformation (Andreev et al., 2007; An et al., 2010; Luo et al., 2017). pHLIP is very practical for targeting acidic tumors, since it can demonstrate three states; *i.e.* soluble in water or bound to membrane surface at normal pH (7.4) or inserted across membrane as an α-helix at low pH (Andreev et al., 2007). Luo *et al.* reported that such a PS could generate ROS upon excitation with laser at 630 nm, resulting in plasma membrane damage and cell death (Luo et al., 2017). Cell membrane-targeting chimeric peptides were also studied by several groups to enhance PDT efficacy (Liu et al., 2017; Ma et al., 2020). Moreover, amphiphilic polymers (Jia et al., 2017; Cai et al., 2018) and fusogenic liposomes (Kim et al., 2017; Bekmukhametova et al., 2020) were shown to insert PSs into cell membrane to increase the therapeutic potential of PDT. In fact, cell membrane-targeted PDT remains infrequently investigated, because of the issues associated with the duration of membrane-anchoring of PSs.

#### Mitochondria-Targeting

Mitochondria are important regulators of apoptosis (Green and Reed, 1998). In general, they are responsible from most of the ATP in a cell. Rubio *et al.* analyzed the spatial dynamics of PDT and reported that mitochondrial targeting is the most efficient PDT in terms of cell killing, while nuclear damage is the least toxic to the cell (Rubio et al., 2009). Therefore, it is reasonable to design and develop PSs that target the mitochondria. PSs can be chemically modified (*e.g.* with triphenylphosphonium derivatives, which insert to the inner membrane of mitochondria) so that they can be actively targeted to mitochondria. Several PSs can accumulate in mitochondria owing to their charge in case of positively charged agents. On the other hand, negatively charged agents accumulate in mitochondria as a result of their hydrophobicity (Morgan and Oseroff, 2001). Furthermore, mitochondrial targeting can also be achieved by synthesizing PSs that are attached to mitochondria targeting sequences, which can direct molecules to the mitochondrial matrix (Murphy, 1997). Hilf showed that porphyrin sensitizers could affect inner mitochondrial membrane enzymes and suggested that mitochondria are important targets in terms of PDT (Hilf, 2007). Thomas *et al.* reported an indocyanine derivative, which showed high mitochondrial targetability. They proposed that mitochondria targeting approach might yield high PDT efficiency (Thomas et al., 2017). Kang and Ko reported that dual selective PDT with a mitochondria targeting PS and fiber optic cannula might prove to be a promising therapy approach (Kang and Ko, 2019). Zhang *et al.* reported a tumor mitochondria specific PDT agent that targets the translocator protein (TSPO) which is localized primarily in the outer mitochondrial membrane (Rupprecht et al., 2010; Zhang et al., 2015). They suggested that such an approach with a translocator protein targeting PS might have a potential (Zhang et al., 2015). Karaman *et al.* reported that mitochondria targeting selenophene modified BODIPY based PSs might prove to be promising in terms of realization of next generation PDT agents (Karaman et al., 2019). In another study, Kessel and Luo utilized 4 PSs with specific targets (mitochondria, lysosomes, plasma membrane) to investigate PDT induced apoptosis (Kessel and Luo, 1998). They reported that PDT caused apoptosis after mitochondrial photodamage (Kessel and Luo, 1998). Several mitochondria targeting moieties (such as triphenylphosphine, guanidinium, bisguanidium) have been reported; however, their efficacies in terms of targeting the mitochondria had not been analyzed in detail. Mahalingam *et al.* prepared triphenylphosphine, guanidinium and bisguanidium derivatives of the verteporfin (a PDT agent approved by the FDA). They reported that mitochondria targeting efficacy of the triphenylphosphine derivative was better. Moreover, it showed better ^1^O_2_ generation and mitochondria membrane toxicity than unmodified verteporfin or its guanidinium derivatives (Mahalingam et al., 2018). Oliveira *et al.* investigated whether subcellular localization may compete with PS efficiency in terms of overall effects of PDT (Oliveira et al., 2011). They proposed that subcellular localization could be more important than photochemical reactivity in terms of PDT. They also suggested that mitochondrial localization might be an important feature in terms of more effective PSs for PDT (Oliveira et al., 2011). In fact, several clinically approved PSs such as Visudyne, Foscan, and Photofrin partially localize to mitochondria (Wilson et al., 1997; Chen et al., 2007a; Celli et al., 2011; Mahalingam et al., 2018). Furthermore, application of aminolevulinic acid results in endogenous synthesis of porphyrins in cells by the heme biosynthetic pathway, generating the main photoactive product PpIX in mitochondria (Gardner et al., 1991).

#### Lysosome-Targeting

In addition to targeting mitochondria, PSs that localize to lysosomes may also demonstrate increased efficacy. Kessel and Reiners Jr suggested that low dose photodamage which sequentially targets lysosomes and mitochondria could provide advantages compared to the use of single PSs (Kessel and Reiners, 2015). Tsubone *et al.* demonstrated that damage in lysosomes was more efficient against HeLa cells than a similar damage in mitochondria (Tsubone et al., 2017). In another study, Li *et al.* reported that ^1^O_2_ generated by a lysosome targeted BODIPY PS could disrupt lysosomes and PDT mediated by that PS could induce apoptosis (Li et al., 2017a). Nguyen *et al.* proposed a new lysosome targeted PS, which showed promising results in terms of PDT (Nguyen et al., 2020). Niu *et al.* reported that a perylene derived PS for lysosome targeting PDT could effectively destruct tumor cells (Niu et al., 2019). Xiao *et al.* suggested that the pyridophenothiazinium dyes might serve as promising lysosome targeting PSs in terms of effective PDT (Xiao et al., 2020). The metallacages that were encapsulated in a polymer to form nanoparticles, which accumulate in the lysosomes, were reported to achieve favorable results in 2-photon PDT (Zhou et al., 2019).

Given all these finding and the vast literature, precise targeting of subcellular organelles may prove to be useful for designing and developing novel effective PDT strategies.

### New Promising Approaches, Enhanced Photodynamic Therapy and Hypoxia

#### Two-Stage Photodynamic Therapy

**FIGURE 7:**
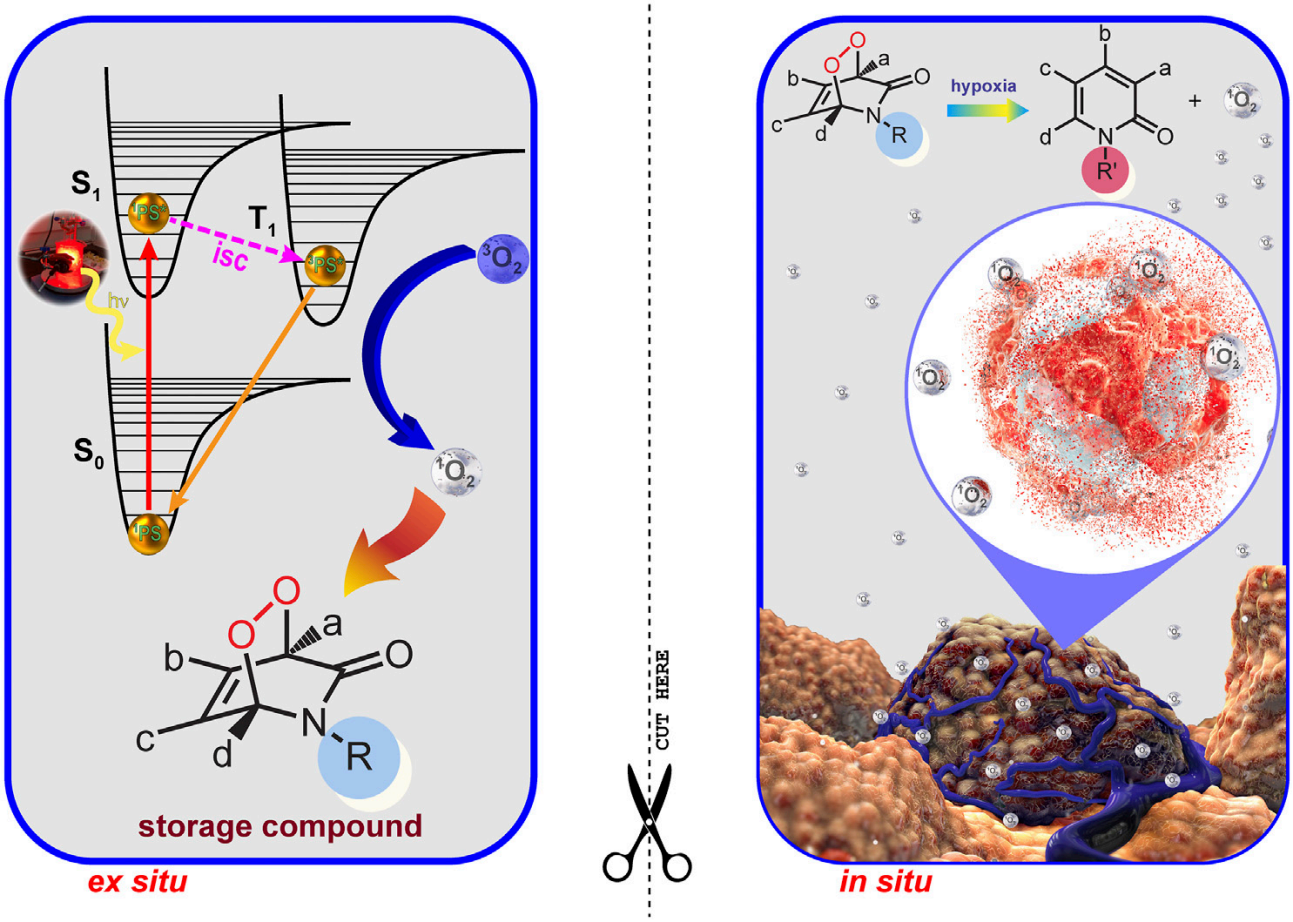
Two Stage PDT. Two stage PDT can overcome the issues of light attenuation and oxygen deficiency in tumors. Reproduced from (Ayan et al., 2020) (published by The Royal Society of Chemistry) with permission from the Royal Society of Chemistry.

Hypoxia is an important feature of most solid tumors. Due to functionally and structurally abnormal microvascular systems, almost all malignant tumors develop hypoxia. From the PDT perspective, hypoxia (oxygen concentrations typically less than 1%) is an insurmountable problem, because molecular oxygen is definitely a fundamental requirement for PDT. Tumor tissues are deprived of oxygen due to their rapid growth-related insufficient vasculature. All these features make hypoxia an important target for therapy. A two-stage PDT (2S-PDT) may overcome the problems of oxygen deficiency and light penetration depth in tumor tissues, which currently limit clinical applications of PDT. In a recent study by Akkaya *et al.*, photosensitization (Stage I) is carried out *ex situ* in the presence of a 2-pyridone derivative and a PS, so the wavelength of excitation becomes unimportant. The endoperoxide product (storage compound) is then transferred to biological conditions. Once triggered by hypoxia, the endoperoxide product was bioreductively changed (Stage II) into a more labile version of itself. Thus, it will specifically generate singlet oxygen, causing “apoptotic response” without depending on, or depleting already low tissue oxygen levels in tumors (Figure 7) (Ayan et al., 2020).

#### Fractional Photodynamic Therapy

**FIGURE 8:**
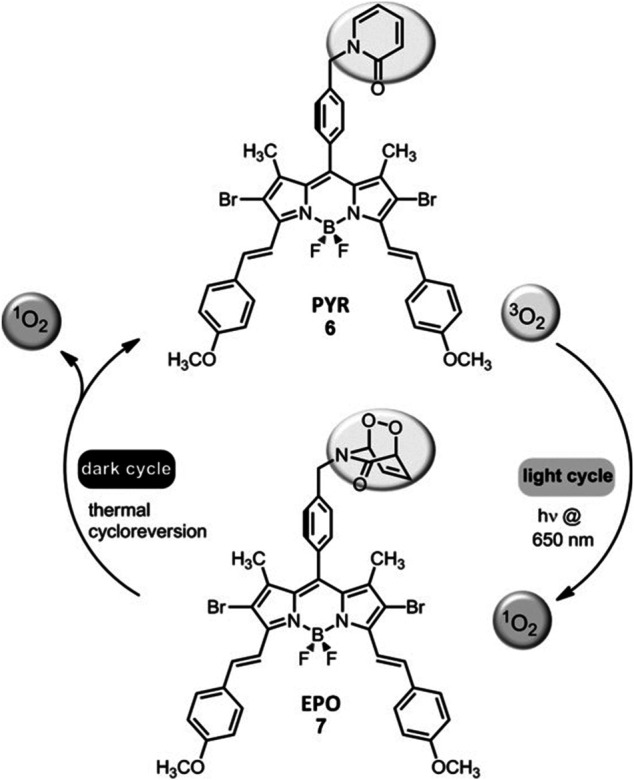
Fractional PDT. Fractional PDT allows for the continuation of photodynamic process both in the dark and in the light cycles (Turan et al., 2016). Reproduced from ref (Turan et al., 2016) with permission from Wiley, copyright 2016.

Tumor tissues are known to have low levels of oxygen. PDT mediated ^1^O_2_ generation *via* PSs in tumors is self-limiting, since the tumor hypoxia may become more severe within tumor tissues during the process. Thus, strategies aimed at reducing photoinduced hypoxia may be beneficial. Accordingly, the light may be introduced intermittently (fractional PDT) in order to allow time for the replenishment of tissue oxygen. Nevertheless, such approaches prolong the period-of-time that is needed for efficient treatment. Akkaya *et al.* showed that a PS with an additional 2-pyridone module to trap ^1^O_2_ could be functional in terms of fractional PDT. In the light cycle, the endoperoxide of 2-pyridone was generated along with ^1^O_2_ (Figure 8). On the other hand, the endoperoxide goes through thermal cycloreversion to produce ^1^O_2_ in the dark cycle. Thus, it regenerates the 2-pyridone module. In summary, photodynamic process can run on both in the dark and light cycles (Turan et al., 2016).

#### Low pH and High Intracellular Glutathione (GSH) Concentration

While energy metabolism in normal cells relies mainly on oxidative phosphorylation and anaerobic glycolysis, energy metabolism in cancer cells relies on aerobic glycolysis (Warburg effect) (Vander Heiden et al., 2009). Cancer cells are constantly exposed to acid-base fluxes due to the high amount of lactic acid resulting from glycolysis. Intracellular pH in cancer cells is stabilized at a favorable level *via* several mechanisms (Swietach et al., 2014).

Tumor microenvironment is slightly acidic and the extracellular pH in tumor tissues is generally lower (6.5–7.2) compared to normal tissues (pH 7.4) (Gerweck and Seetharaman, 1996). This feature may be utilized in order to target tumor tissues. Oxidative/metabolic stress increases in cancer cells. In this context, GSH, which plays an essential role in antioxidant systems, increases in cancer cells (Traverso et al., 2013; Kennedy et al., 2020). Thus, a high GSH level may be a crucial cancer-specific target (Li et al., 2017c). In a study by Akkaya *et al.*, a series of pH and GSH responsive PSs were designed. pK_a_ values were optimized to reach a pH range of slight acidity (6.0–7.4). pH-activatable behavior and redox mediated release of the quencher from the PS by GSH allowed for selective PDT.

#### Multimodal Synergistic Therapies

Combination of PDT with other therapy modalities may be promising in order to increase effectiveness against tumors (Firczuk et al., 2011). Dual or tri-modal synergistic therapy approaches such as SDT (sonodynamic therapy), CT (chemotherapy), RT (radiotherapy) and immunotherapy enhance the effectiveness of PDT (Allman et al., 2000; Fan et al., 2016; Lan et al., 2019). Combination of PDT with chemotherapy can achieve increased tumoricidal effects (Nahabedian et al., 1988).

Several sensitizers were reported to be activated by ultrasound, instead of light. Although the tissue penetration of ultrasound utilized in SDT strategy is higher than UV-VIS used in PDT, sensitizer diversity is quite limited (Qian et al., 2016). For this reason, SDT and PDT synergistic treatment strategy may offer a more effective treatment than monotherapy (Abrahamse and Hamblin, 2016). In addition, sono/photodynamic combined therapy approaches were reported to improve the efficacy of various immunotherapeutic strategies used in tumors (Shi et al., 2019; Li et al., 2020b). PDT can trigger immunogenic cell death, which stimulates immune responses against dead cell antigens (Green et al., 2009; Kroemer et al., 2013; Ng et al., 2018). Indeed, combination of PDT with cancer immunotherapy can show synergistic results, cause tumor regression and achieve immune memory (Ng et al., 2018).

## Concluding Remarks

PDT has a favorable adverse event profile, demonstrate minimally invasive characteristics and is able to enhance anti-tumor immune responses. Although the earliest preclinical applications of PDT were published more than a century ago and Photofrin received the FDA approval in 1995, it is disappointing that PDT is not broadly utilized in the clinical setting for the treatment of malignant and/or non-malignant diseases. In fact, we have witnessed great developments in terms of illumination techniques, nanotechnology, smart chemical designs as well as understanding the biological mechanisms implicated in the responses to PDT. Pioneering approaches in designing and synthesizing novel PSs demonstrated promising preclinical outcomes. Moreover, the role and effectiveness of PDT in treatment of various diseases have been investigated. However, several issues still hinder the development of PDT, such as limitations related with light, tissue oxygenation and inherent properties of the PSs (*e.g.* water solubility). In addition, the mechanisms of action of PDT seem to be not completely understood. PDT is capable of inducing apoptosis, necrosis as well as autophagy; and these mechanisms can be activated simultaneously. Thus, a thorough insight into photobiological and photochemical mechanisms seems to be priceless for designing novel effective PDT strategies. Furthermore, several studies reported inconsistent clinical results. Thus, we still need to improve PDT strategies and perform clinical studies in order to demonstrate the efficacy of PDT in comparison to other treatment modalities such as surgery and chemotherapy. In order to overcome the current problems and widen the applications of PDT, strategies aimed to solve issues associated with light and drug dose seem to be crucial. Exceptional designs that increase the selectivity of PDT will also assume indispensable roles. In addition, subcellular/organelle targeting strategies have the potential to improve the effectiveness of PDT. Novel concepts in the field (*e.g.* enhanced, two-stage, fractional PDT strategies) may also prove to be very useful for pursuing and improving effective PDT strategies. Finally yet importantly, the therapeutic application methods of PDT as well as their ease-of-use should also be considered carefully, so that clinical expansion of PDT can be achieved. In this respect, combining PDT with other treatment options such as chemotherapy and immunotherapy may indeed yield better results. Such combination strategies underline the importance of clinical studies which investigate the effectiveness of multimodal therapy approaches that incorporate PDT. It will be undoubtedly exciting to see future innovative studies that aim to overcome the limitations and reveal the full potential of PDT in terms of clinical translation.

## Abbreviations

ALA, Aminolevulinic acid; AMD, Age related macular degeneration; ATG, Autophagy-related protein; atp, Atmospheric pressure; ATPL, Antibody-targeted photolysis; BODIPY, 4,4-difluoro-4-bora-3a,4a-diaza-s-indacene; BRET, Bioluminescence resonance energy transfer; CNV, Choroidal neovascularization; COVID-19, Coronavirus disease 2019; CRET, Čerenkov resonance energy transfer; CT, Chemotherapy; DAR, Darier’s disease; DL, Daylight; DMA, 2,3-dimethylmaleic anhydride; EPR, Enhanced permeability and retention; FDA, Food and Drug Administration; FRET, Förster resonance energy transfer; GFP, Green fluorescent protein; GSH, Glutathione; HBO_2_, Hyperbaric oxygen; HpD, Hematoporphyrin derivative; ICAM, Intracellular adhesion molecule; IL, Interleukin; *i.v*, intravenous; JNK, c-Jun N-terminal kinases; LED, Light emitting diodes; mAbs, Monoclonal antibodies; MAPK, Mitogen-activated protein kinases; mPDT, Metronomic photodynamic therapy; MSN, Mesoporous silica nanoparticles; *m*-THPC, *meta*-tetrahydroxyphenylchlorin; mTOR, Mammalian target of rapamycin; NIR, Near-infrared; NK, Natural killer; NP, Nanoparticles; PDR, Photodynamic reaction; PDT, Photodynamic therapy; PGE2, Prostaglandin E2; PIT, Photoimmunotherapy; p.o, per os; PpIX, Protoporphyrin IX; PS, Photosensitizer; RIP, Receptor-interacting protein; ROS, Reactive oxygen species; RT, Radiotherapy; SDT, Sonodynamic therapy; TNF, Tumor necrosis factor; TSPO, Translocator protein; UCN, Upconversion nanoparticle; VEGF, Vascular endothelial growth factor; VP, Verteporfin.
